# Epidemiology and In Vitro Activity of Ceftazidime/Avibactam, Meropenem/Vaborbactam and Imipenem/Relebactam against *KPC*-Producing *K. pneumoniae* Collected from Bacteremic Patients, 2018 to 2020

**DOI:** 10.3390/antibiotics11111621

**Published:** 2022-11-14

**Authors:** Federica Bovo, Donatella Lombardo, Tiziana Lazzarotto, Simone Ambretti, Paolo Gaibani

**Affiliations:** Microbiology Unit, IRCCS Azienda Ospedaliero-Universitaria di Bologna, 40138 Bologna, Italy

**Keywords:** BL-BLICs, resistance, enterobacteriales, whole-genome sequencing

## Abstract

The management of KPC-producing *K. pneumoniae* (KPC-Kp) in bloodstream infections (BSIs) represent a serious clinical challenge. In this study, the aim is to assess the incidence of resistance to novel β-lactams-β-lactamase inhibitor combinations (βL-βLICs), such as ceftazidime-avibactam (CAZ-AVI), meropenem-vaborbactam (MER-VAB) and imipenem-relebactam (IMI-REL), in KPC-Kp strains collected during a three-year period from patients with bacteremia. KPC-Kp strains resistant to βL-βLICs were selected for whole-genome sequencing. A total of 133 *K. pneumoniae* strains were isolated, and KPC-Kp strains were the most represented (87.2%). In 2018, resistance to CAZ-AVI and MER-VAB was 6.5% and 14.5%, respectively. In 2019, KPC-Kp resistance to CAZ-AVI and MER-VAB remained at low levels, with values of 12.9% and 3.2%, respectively. During 2020, CAZ-AVI resistance was detected in 2/23 of KPC-Kp strains (8.7%). IMI-REL was the most active βL-βLIC, inhibiting >98% of the isolates, while CAZ-AVI and MER-VAB inhibited 87–93% and 85–97% of the KPC producers, respectively. Correlations between genotypic traits and resistance to βL-βLICs showed that KPC-Kp strains resistant to CAZ-AVI harbored a mutation within the *bla*_KPC-3_ gene, while all KPC-Kp strains resistant to CAZ-AVI, MER-VAB and/or IMI-REL carried the *bla*_KPC-3_ gene. Moreover, genetic analysis of porin genes showed that 14/16 of KPC-Kp resistant isolates possessed a truncated OmpK35 and glycine (G) and aspartic acid (D) insertions at positions 134–135 within OmpK36, whereas 2/16 displayed truncated OmpK35 and OmpK36 porins. Novel βL-βLICs are promising agents against KPC-Kp infections; however, the emergence of resistance to these agents highlights the need for continuous surveillance and application of enhanced antimicrobial stewardship.

## 1. Introduction

During the last two decades, multi-drug-resistant (MDR) gram-negative bacteria have represented a serious public health concern due to the reduced availability of antimicrobial options associated with increased morbidity and mortality as well as higher healthcare costs. According to the World Health Organization (WHO), carbapenemase-resistant *Enterobacterales* (CRE) represent a highly critical group of MDR organisms for which new treatments are needed [1,2,3]. Among CRE, *Klebsiella pneumoniae* represents one of the most clinically relevant pathogens [4] for nosocomial and community acquired infections [5]. Resistance to carbapenem in *K. pneumoniae* is due to different mechanisms, including the production of KPC-type class A carbapenemases [4] and/or additional mechanisms, such as a lack of porin functionality and up-regulation of the efflux system [6]. At the same time, KPC carbapenemase production represents the most relevant mechanism in clinical practice due to the limited availability of molecules with in vitro activities [6]. In recent years, βL-βLICs have been approved for treatment of infections due to CRE [7,8] and have represented the main strategy against KPC-mediated resistance [9]. Avibactam, vaborbactam and relebactam are newer inhibitory agents with a high affinity for Ambler class A β-lactamases (extended-spectrum β-lactamases [ESBLs], KPCs) and C (e.g., Amp C) and with favorable outcomes in current clinical trials [10]. CAZ-AVI was the first βL-βLIC approved in 2015 by the FDA for complicated intra-abdominal infections (cIAIs), complicated urinary tract infections (cUTIs) and, subsequently, for the treatment of hospital-acquired and ventilator-associated bacterial pneumonia (HABP/VABP) [11]. Although CAZ-AVI is highly effective against class A, C and D β-lactamases, resistance to CAZ-AVI emerged rapidly in *Enterobacterales* due to specific mutations within class A carbapenemases, which are the most common mechanisms related to an increase in the minimum inhibitory concentration (MIC) values for CAZ-AVI, in combination with the modification of the antibiotic target and changes in cell permeability [12]. MER-VAB was approved in 2017 by the FDA for the treatment of cUTIs, cIAIs and HABP/VABP [13]. In vitro studies demonstrated that vaborbactam reduced the MIC values of meropenem by ≥ 64-fold against carbapenem-resistant strains producing KPC [14]. However, recent studies have demonstrated that the emergence of KPC-producing *Enterobacterales* resistant to MER-VAB was related to impaired permeability, due to the loss of expression of porins, and was associated with the increase in copies of the *bla*_KPC_ genes and activation of efflux pumps [15]. IMI-REL was approved by the FDA in 2019 for the treatment of cIAIs, cUTIs and, subsequently, HABP/VABP [16]. Lately, resistance to IMI-REL in KPC-Kp strains correlated with mutations in OmpK35/36 porins, joined with the hyper-production of KPC-β-lactamases and efflux pump down-regulation, has been observed [17,18]. The development of resistance to these new drugs has been already described, and the emergence of resistance to these agents highlights the critical impact in choosing among treatment options [10,19]. Hence, in this study, we evaluated the incidence of resistance against CAZ-AVI, MER-VAB and IMI-REL in KPC-Kp strains isolated from patients with bacteremia. 

## 2. Results 

A total of 133 *K. pneumoniae* meropenem-resistant strains were isolated between January 2018 and December 2020. In particular, 116 (87.2%) were KPC, while 17 harbored different types of carbapenemases, which were thus distributed: 13 NDM (9.8%), 3 VIM (2.3%) and 1 OXA48 (0.7%). During 2018, antimicrobial susceptibility tests of KPC-Kp isolates showed that 93,5% (58/62) were susceptible to CAZ-AVI, while resistance was revealed in 6.5% (4/62) of KPC-Kp isolates (Table 1). Susceptibility to MER-VAB was 85.5% (53/62), while resistance was displayed in 14.5% (9/62) of KPC-Kp isolates. Moreover, susceptibility to IMI-REL was detected in 98.4% (61/62) of KPC-Kp strains. Three KPC-Kp strains showed cross-resistance to CAZ-AVI and MER-VAB, while only one KPC-Kp strain showed cross-resistance to CAZ-AVI, MER-VAB and IMI-REL. During 2019, the distribution of resistance to βL-βLICs in KPC-Kp strains did not indicate any statistically significant difference from 2018 to 2019. In particular, antimicrobial susceptibility tests of KPC-Kp isolates showed that 87.1% (27/31) were susceptible to CAZ-AVI, while resistance was revealed in 12.9% (4/31) of KPC-Kp isolates. Susceptibility to MER-VAB and IMI-REL was 96.8% (30/31), while resistance was displayed in 3.2% (1/31) of KPC-Kp isolates. One KPC-Kp strain showed cross-resistance to CAZ-AVI, MER-VAB and IMI-REL. In 2020, during the COVID-19 pandemic, 23 KPC-Kp strains isolated from BSIs were analyzed. Antimicrobial susceptibility tests showed that 91.3% (21/23) were susceptible to CAZ/AVI, while resistance was detected in 8.7% (2/23) of KPC-Kp strains. Cross-resistance to MER-VAB and IMI-REL was detected in one KPC-Kp strain, thus revealing a sensitivity towards KPC-Kp strains of 95.6% (22/23).

MALDI-TOF analysis conducted on CAZ-AVI and/or MER-VAB and/or IMI-REL -resistant strains showed that 4 out of 16 (25%) KPC-Kp harbored the 11.109-Da peak related to the bla_KPC_ allele (data not shown). Genomic analysis performed on 16 KPC-Kp strains resistant to CAZ-AVI and/or MER-VAB and/or IMI-REL (Table 2) belonged to three different sequence types (ST) including ST512, ST1519 and ST307 (Table 3). Resistome analysis demonstrated that all strains exhibited similar genetic resistance determinants responsible for resistance to the same drug combination. In particular, analysis of β-lactams resistance genes showed that eight out of nine KPC-Kp resistant strains collected during 2018 carried the *bla*_KPC-3_ and *bla*_SHV-182_ genes and were resistant to MER-VAB or MER-VAB and CAZ-AVI, while one out of nine was resistant to CAZ-AVI, MER-VAB and IMI-REL, harboring the *bla*_KPC-3_ and *bla*_SHV-11_ genes. In 2019, three out of four KPC-Kp resistant strains collected showed resistance to CAZ-AVI and harbored the *bla*_KPC-3/31_, *bla*_TEM-1_ and *bla*_SHV-11/28_ gene variants, while the one out of four was resistant to CAZ-AVI, MER-VAB and IMI-REL and showed the *bla*_KPC-3_ and *bla*_SHV11_ genes. During 2020, two KPC-Kp strains resistant to CAZ-AVI were detected, one carrying the *bla*_KPC-31_, *bla*_TEM-1_ and *bla*_SHV-28_ genes and the other carrying the *bla*_KPC-86_, *bla*_TEM-128_ and *bla*_SHV-11_ resistance genes. In addition, one KPC-Kp strain showed resistance to MER-VAB and IMI-REL, carrying the *bla*_KPC-3_, *bla*_TEM-1_ and *bla*_SHV-11_ variants. Genetic analysis of porin genes showed that 14 out of 16 KPC-Kp isolates possessed a truncated OmpK35 and glycine (G) and aspartic acid (D) insertions at positions 134–135 within OmpK36. At the same time, 2 out of 16 CAZ-AVI-resistant KPC-Kp strains displayed truncated OmpK35/36 porin proteins (Table 3). 

Correlations between genotypic traits and resistance to βL-βLICs showed that four out of five KPC-Kp strains resistant to CAZ-AVI harbored a mutated *bla*_KPC_ (Table 3). In particular, CAZ-AVI resistant strains carried the *bla*_KPC-31_ and *bla*_KPC-86_ genes. At the same time, all KPC-Kp strains resistant to MER-VAB or IMI-REL carried the *bla*_KPC-3_ gene. Lastly, KPC-Kp strains resistant to CAZ-AVI, MER-VAB and/or IMI-REL carried the *bla*_KPC-3_ gene. 

**Figure 1:**
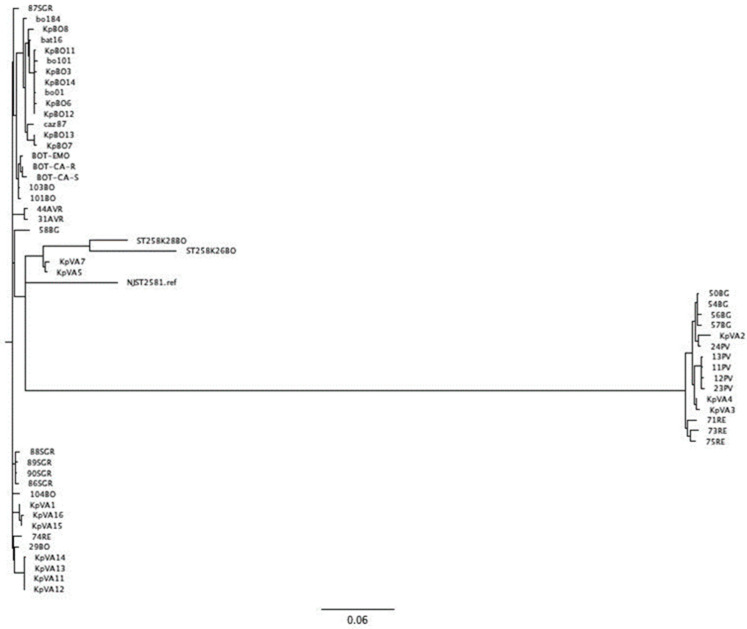
Phylogenetic tree analysis of KPC-Kp Italian isolates and strains included in this study.

To evaluate the relationship between KPC-Kp strains collected during the study, we performed a phylogenetic analysis using the SNPs of KPC-Kp genomes within the core genomes. Our analysis showed that all strains included in this study belonging to the Clonal Complex (CC258) clustered closely (Figure 1). At the same time, strains belonging to the ST307 segregated separately from the CC258 (data not shown).

## 3. Discussion 

In this study, we analyzed the phenotypic and genotypic characteristics of KCP-Kp strains isolated from patients with bacteremia, detected during routine susceptibility test screening. The number of KPC-Kp isolates detected during the three-year period decreased from 62 in 2018 to 23 in 2020. During 2019, CAZ-AVI resistance rates in KPC-Kp isolates remained low, ranging from 6.5% (4/62) detected in 2018 to 12.9% (4/31). Recent hospitalization abroad is the main risk factor for the acquisition of KPC, and data from 2020 were not representative of the usual epidemiology of carbapenem susceptibility to KPC due to travel restrictions and downscaling of non-urgent healthcare procedures caused by the COVID-19 pandemic. All new βL-βLICs inhibited >85% of KPC-Kp isolates. More specifically, IMI-REL displayed the greatest activity, inhibiting >98% of KPC-Kp isolates at ≤1.5 μg/mL, following the current EUCAST breakpoints, while CAZ-AVI and MER-VAB inhibited approximately 87–93% and 85–97% of the isolates, respectively. 

Sequencing the resistant KPC-Kp strains revealed that mutations in the *bla*_KPC-3_ gene are involved in resistance to CAZ-AVI [20], while resistance to MER-VAB in KPC-3-producing *K. pneumoniae* strains is mostly associated with loss-of-function in the OmpK36 porin protein [21]. Finally, IMI-REL resistance is linked to the concomitant increase in MIC values for MER-VAB and CAZ-AVI, suggesting that cross-resistance could represent a mechanism for the onset of resistance between novel βL-βLICs [22]. 

This study is a later work, following the analysis of the epidemiology of KPC-Kp strains isolated in our hospital during 2018 [23], and is not completely representative of the large-scale epidemiological trend as it includes a limited number of patients enrolled. Therefore, the emergence of resistance during therapy has already been observed against CAZ-AVI and MER-VAB [10], and the presence of different resistance mechanisms observed in KPC-Kp strains makes it difficult to predict the susceptibility profiles of βL-βLICs. In light of current clinical evidence, the new components remain effective as an additional option for the treatment of infections caused by KPC-Kp. 

In conclusion, it is reasonable to assume that all new antibiotics should be used with caution and always within a well-defined antimicrobial management program.

## 4. Materials and Methods 

### 4.1. Study Participants

Between January 2018 and December 2020, we collected KPC-Kp strains isolated from adult patients with positive blood cultures hospitalized at the Policlinico di Sant’Orsola (PSO), a large tertiary-care university hospital in Bologna. The PSO is a 1420-bed university hospital with an average of 72,000 admissions per year. Patients were included only during their first episode of BSI due to KPC-Kp and were kept anonymous throughout the study, following the Declaration of Helsinki guidelines and its later amendments. 

### 4.2. Phenotypic Analysis

*Klebsiella pneumoniae* isolates recovered from the blood samples of patients were collected between January 2018 and December 2020 during routine active surveillance screenings at the Microbiology Unit of S. Orsola-Malpighi Hospital (Bologna, Italy). The blood samples were processed following the routine workflow of the microbiology laboratory of Sant’Orsola-Malpighi University Hospital. After the Gram staining examination, positive blood cultures were plated on horse blood agar and CHROMagar Orientation, to ensure pure, isolated colonies, and were subsequently identified using matrix-assisted laser desorption/ionization time-of-flight mass spectrometry (MALDI-TOF MS) (Bruker Daltonics, Leipzig, Germany). Antimicrobial susceptibility tests were performed using MicroScan Walkaway-96 (Beckman Coulter, Brea, California, US). The minimal inhibitory concentration (MIC) values for CAZ-AVI, MER-VAB and IMI-REL were tested using MIC test strips (Liofilchem, Roseto degli, Abruzzi, Italy). MIC values were interpreted using EUCAST breakpoints v12.0 (available at: http://www.eucast.org/clinical_breakpoints/, accessed on 1 January 2020). *Enterobacterales* exhibiting carbapenem-resistance were screened for carbapenemase production and typing following routine workflow [24,25]. Briefly, specific peak (11.109 m/z) detection analysis for KPC-type carbapenemases [26,27] was used as a first-line detection assay followed by an immunochromatographic assay using NG-Test CARBA 5 (NG Biotech, Guipry, France). In case of discordant results, the presence of a carbapenemase gene was confirmed with a molecular assay using Xpert Carba-R (Cepheid, Sunnyvale, CA, USA). 

### 4.3. Whole Genome Analysis

Strains exhibiting resistance to CAZ-AVI and/or MER-VAB and/or IMI-REL were selected for whole-genome sequencing. Genomic DNA was extracted from purified cultures of *Klebsiella pneumoniae* using DNeasy Blood&Tissue Kit (Qiagen, Basel, Switzerland), following the manufacturer’s instructions, and was further cleaned with AMPure XP magnetic beads (Beckman Coulter, Krefeld, Germany). Whole-genome analysis was performed as previously described [28]. Briefly, bacterial genomes were sequenced using the Illumina iSeq 100 platform (iSeq Reagent Kit v2, Illumina, San Diego, CA, USA) with an iSeq Reagent kit v2 and 2 × 150 paired-end reads after using Illumina DNA Prep paired-end library preparation. Read sets were evaluated for sequence quality and read-pair length using FastQC software (available at: https://www.bioinformatics.babraham.ac.uk/projects/fastqc/, accessed on 9 October 2022). Genome assemblies were performed using SPAdes v.3.10 with careful settings and were polished with Pilon v.1.23. Annotation was automatically carried out using RAST server (available at: https://rast.nmpdr.org, accessed on 9 October 2022) and manually curated using Artemis v.17.0.1. Antimicrobial resistance genes, plasmid content and MLST analysis were assessed using an online platform (available at: https://cge.cbs.dtu.dk/services/, accessed on 9 October 2022). ß-lactamase content was confirmed using BLAST analysis against CARDB and Beta-Lactamase-Database (http://bldb.eu, accessed on 9 October 2022). Porin genes were manually investigated using BLAST analysis against a reference protein (OmpK35 [O87753], OmpK36 [D6QLX8] and OmpK37 [S5UDN6]), and prophage regions within the KPC-Kp genome were assessed using PHASTER (https://phaster.ca, accessed on 9 October 2022). The phylogenetic tree was generated using core genome SNP analysis as previously described [28]. SNPs and insertion-deletions (Indels) between CAZ-AVI and/or MER-VAB and/or IMI-REL resistant genomes were investigated as previously described [22,28].

Genomes included in this study were deposited in GenBank under the following accession numbers: (BO1) SAMN31571291, (BO3) SAMN18746645, (BO6) SAMN18746646, (BO7) SAMN18746647, (BO8) SAMN18746648, (BO11) SAMN18746649, (BO12) SAMN18746650, (BO13) SAMN18746651, (BO14) SAMN18746652, (BO77) SAMN31571292, (BO101) SAMN31571293, (BO184) SAMN31571294, (BO204) SAMN31571295, (BO302) SAMN26209580, (BO628) SAMN28544955, (BO630) SAMN28544957.

## Figures and Tables

**Table 1:** Three-year trend of resistance to βL-βLICs in KPC-Kp strains isolated from BSIs.

Antimicrobial Agent	% Resistance ^a^			
	Total of KPC-Kp Isolates (*n* = 116)	2018 (*n* = 62)	2019 (*n* =31)	2020 (*n* = 23)
Ceftazidime-avibactam	8.6% 10/116	6.5% (4/62)	12.9% (4/31)	8.7% (2/23)
Meropenem-vaborbactam	9.5% 11/116	14.5% (9/62)	3.2% (1/31)	4.3% (1/23)
Imipenem-relebactam	2.6% 3/116	1.6% (1/62)	3.2% (1/31)	4.3% (1/23)

^a^ Applying EUCAST breakpoints.

**Table 2:** Phenotypic characteristics of KPC-Kp strains included in this study.

Isolate	Year	MIC (mg/L)		
		CAZ-AVI	MER-VAB	IMI-REL
**BO1**	2018	**16**	**16**	**8**
BO3	2018	**16**	**>256**	1.5
BO6	2018	**16**	**256**	0.38
BO7	2018	**256**	**256**	1
BO8	2018	6	**48**	1
BO11	2018	8	**256**	1
BO12	2018	8	**256**	0.5
BO13	2018	6	**256**	0.5
BO14	2018	8	**256**	1.5
BO77	2019	**>256**	<0.06	0.12
BO101	2019	**>256**	**>256**	**>8**
BO184	2019	**16**	8	2
BO204	2019	**>256**	2	1
BO302	2020	8	**16**	**4**
BO628	2020	**>256**	0.032	0.094
BO630	2020	**32**	1	0.25

Resistance values are shown in bold.

**Table 3:** Genotypic characteristics of KPC-Kp strains included in this study.

Isolate	ST	Carbapenemase	β-Lactamases	Porins		Plasmid
				OmpK35	OmpK36	
BO1	512	*bla*_KPC-3_	*bla*_SHV-11_	truncated at aa 42	INS135GD	ColRNAI, IncFIB(K), IncFIB(pKPHS1), IncX3
BO3	512	*bla*_KPC-3_	*bla*_SHV-182_	truncated at aa 41	INS135GD	ColRNAI, IncFIB(K), IncFIB(pKPHS1), IncX3
BO6	512	*bla*_KPC-3_	*bla*_SHV-182_	truncated at aa 41	INS135GD	ColRNAI, IncFIB(K), IncFIB(pKPHS1), IncX3
BO7	1519	*bla*_KPC-3_	*bla_TEM-1A_, bla*_SHV-182_, *bla_OXA-9_*	truncated at aa 41	INS135GD	ColRNAI, IncFIB(K), IncFIB(pKPHS1), IncFIB(pQil), IncFII(K), IncX3
BO8	512	*bla*_KPC-3_	*bla_TEM-1A_, bla*_SHV-182_, *bla_OXA-9_*	truncated at aa 41	INS135GD	ColRNAI, IncFIB(K), IncFIB(pKPHS1), IncFIB(pQil), IncFII(K), IncX3
BO11	512	*bla*_KPC-3_	*bla*_SHV-182_	truncated at aa 41	INS135GD	ColRNAI, IncFIB(K), IncFIB(pKPHS1), IncFII(K), IncX3
BO12	512	*bla*_KPC-3_	*bla*_SHV-182_	truncated at aa 41	INS135GD	ColRNAI, IncFIB(K), IncFIB(pKPHS1), IncFII(K), IncX3
BO13	1519	*bla*_KPC-3_	*bla*_SHV-182_, *bla_OXA-9_*	truncated at aa 41	INS135GD	ColRNAI, IncFIB(K), IncFIB(pKPHS1), IncFIB(pQil), IncFII(K), IncX3
BO14	512	*bla*_KPC-3_	*bla*_SHV-182_	truncated at aa 41	INS135GD	ColRNAI, IncFIB(K), IncFIB(pKPHS1), IncX3
BO77	307	*bla*_KPC-31_	*bla_TEM-1_, bla*_SHV-28,_ *bla_OXA-1_*	truncated at aa 229	truncated at aa 182	IncFIB(K), IncFIB(pQil), IncFII(K)
BO101	512	*bla*_KPC-3_	*bla*_SHV-11_	truncated at aa 42	INS135GD	ColRNAI, IncFIB(K), IncFIB(pKPHS1), IncX3
BO184	512	*bla*_KPC-3_	*bla_TEM-1_, bla*_SHV-11_	truncated at aa 42	INS135GD	ColRNAI, IncFIB(K), IncFIB(pKPHS1), IncFIB(pQil), IncFII(K)
BO204	512	*bla*_KPC-31_	*bla_TEM-1_, bla*_SHV-11_	truncated at aa 42	INS135GD	IncFIB(K), IncFIB(pKPHS1), IncFIB(pQil), IncFII(K), IncX3
BO302	512	*bla*_KPC-3_	*bla_TEM-1_, bla*_SHV-11_	truncated at aa 41	INS135GD	ColRNAI, IncFIB(K), IncFIB(pKPHS1),IncFIB(pQil), IncFII(K), IncX3
BO628	307	*bla*_KPC-31_	*bla_TEM-1_, bla*_SHV-28_, *bla*_CTX-M-15_	truncated at aa 229	truncated at aa 182	IncFIB(K), IncFIB(pQil), IncFII(K)
BO630	1519	*bla*_KPC-86_	*bla_TEM-128_, bla*_SHV-11_	truncated at aa 41	INS135GD	Col(BS512), ColRNAI, IncFIB(pKPHS1), IncFIB(pQil), IncFII(K)
